# Clinicopathologic features of superficial esophageal squamous cell carcinoma in different infiltrative growth pattern

**DOI:** 10.3389/fonc.2025.1512433

**Published:** 2025-07-30

**Authors:** Xing Qi, Zhenxiang Zuo, Bin Yu, Huimin Zhang, Xiujie Cui, Guangchun Li, Honglei Wu

**Affiliations:** ^1^ Department of Gastroenterology, The Second Hospital, Cheeloo College of Medicine, Shandong University, Jinan, Shandong, China; ^2^ Department of Hematology, The Second Hospital, Cheeloo College of Medicine, Shandong University, Jinan, Shandong, China; ^3^ Department of Pathology, The Second Hospital, Cheeloo College of Medicine, Shandong University, Jinan, Shandong, China

**Keywords:** SESCC, INF, IPCL, infiltration depth, clinicopathologic features

## Abstract

**Introduction:**

Superficial esophageal squamous cell carcinoma (SESCC) is defined as neoplastic lesions limited to the mucosa or submucosa regardless of the nodal status. The infiltrative growth pattern (INF) has been implicated in tumor aggressiveness and prognosis in various cancers, but the application research of INF in SESCC is still unclear. We aimed to investigate the association between INF types and clinicopathological features in SESCC.

**Methods:**

We retrospectively analyzed 368 SESCC patients who underwent endoscopic submucosal dissection and precisely defined INF classification from 2014 to 2023. INF was classified as INFa/b/c per Japanese Esophageal Society guidelines. Clinicopathological characteristics were compared across INF types using univariate analysis. Significant variables from univariate analysis were included in multivariate logistic regression to identify independent predictors of INF types.

**Results:**

Univariate analysis revealed that the INF of tumor was associated with tumor size, gross morphology, intraepithelial papillary capillary loop (IPCL), infiltration depth, lymphovascular invasion, and vertical positive margins (All *P* < 0.05). Multivariate logistic regression demonstrated that tumor size (*β*=0.28, P=0.004; OR=1.32, 95%CI:1.09-1.59), IPCL (*β*=0.81, P=0.004; OR=2.24, 95%CI:1.30-3.85), and infiltration depth (*β*=0.81, P=0.017; OR=2.24, 95%CI:1.15-4.35) were significantly correlated with INFb, while lymphovascular invasion (*β*=8.77, P=0.007; OR=6456.93, 95%CI:10.96-3803785.49) as an independent risk factor for INFc.

**Conclusion:**

Increased tumor size, presence of IPCL type B2, and depressed gross morphology were more indicative of INFc-type SESCC. Compared with INFa and INFb, INFc type SESCC has deeper infiltration depth and is more likely to have lymphovascular invasion and positive postoperative resection margins. Therefore, careful endoscopic visualization of tumor size, IPCL, and gross morphology can improve the prediction of INF and tumor status, facilitating informed preoperative selection of surgical approach and subsequent postoperative treatments.

## Introduction

Esophageal cancer (EC) is a kind of malignant tumor that seriously threatens human health, that often is diagnosed in advanced stages, resulting in a poor prognosis (1). It primarily consists of two major subtypes: esophageal squamous cell cancer (ESCC) and esophageal adenocarcinoma (EAC) (2, 3). The treatment approaches for esophageal cancer differ significantly depending on the pathological type and clinical stage in cases of the same type (4). Although neoadjuvant therapy and other treatment methods benefit patients with advanced esophageal cancer (5), early detection and treatment are key to improving the survival rate and the quality of life of esophageal cancer.

Superficial esophageal squamous cell carcinoma (SESCC) is defined that the neoplastic lesions limited to the mucosa or submucosa regardless of the nodal status (1). At present, endoscopic resection (ER) is preferred for SESCC for medically fit patients. Therefore, it is of great significance to clarify the clinical endoscopy and pathological features of SESCC.

According to the Japanese classification of esophageal cancer, the infiltrative growth pattern (INF) indicates the growth and infiltrative pattern of tumors (2). INF has been reported as a prognostic factor for stomach, bladder, and other cancers. Moreover, INF was also reported as a prognostic factor correlating with tumor depth and lymphatic invasion in superficial esophageal cancer (3, 6, 7). However, the application research of INF in SESCC is still unclear. In Japan, the INF has been routinely assessed as a pathologic characteristic of surgically resected specimens. However, it has not been gained widespread use in the clinic (8).

In this study, we focused on the tumor INF type and clinicopathological characteristics of SESCC. We analyzed a series of 368 patients with a diagnosis of SESCC and clearly define the INF classification who underwent ESD. Compared with INFa and INFb, the INFc type SESCC has a deeper infiltration depth and is more prone to lymphovascular invasion. In addition, the INFc type SESCC is more likely to have a positive postoperative resection margin. Therefore, the INF type can be applied in the histopathological assessment of endoscopically resected specimens of SESCC, and better to predict the more possibility of tumor metastasis.

In conclusion, the careful visualization of tumor size, intraepithelial papillary capillary loop (IPCL), gross morphology and INF under endoscopy, could roughly predicted the tumor, which helps to improve the accuracy of biopsy and achieve the purpose of guiding the rational selection of surgical methods. Moreover, improving the prediction of INF and tumor status of SESCC is more conducive to the wise selection of surgical approaches and help for the selection of more aggressive postoperative treatment.

## Materials and methods

### Patient selection

This study enrolled 368 patients diagnosed with SESCC and clearly defined INF classification who underwent endoscopic submucosal dissection (ESD) between 2014 and 2023. The clinical and pathological data were obtained through a detailed retrospective review of the medical records. The study protocol was approved by the ethics committee of the second hospital of Shandong university, China (Reference number KYLL2024728).

### Tissue preparation and evaluation of histopathological factors

The resected specimens were fixed on a cork board with the mucosal membrane extended. Serial sections of 4 μm were cut from the specimens, fixed with 10% buffered formalin, and stained with Hematoxylin and Eosin (H&E). All available H&E-stained tumor slides were reviewed by two pathologists separately who were blinded to clinical data at the time of the histologic evaluation. Whenever there was uncertainty, a third experienced pathologist was consulted. The invasive depth of SESCC was subclassified into 3 groups: lamina propria mucosa (M2), muscularis mucosa (M3) and superficial layer of the submucosa (SM1), middle layer of the submucosa (SM2) and deeper layer of the submucosa (SM3).

### Microscopic evaluation of the INF

**Figure 1:**
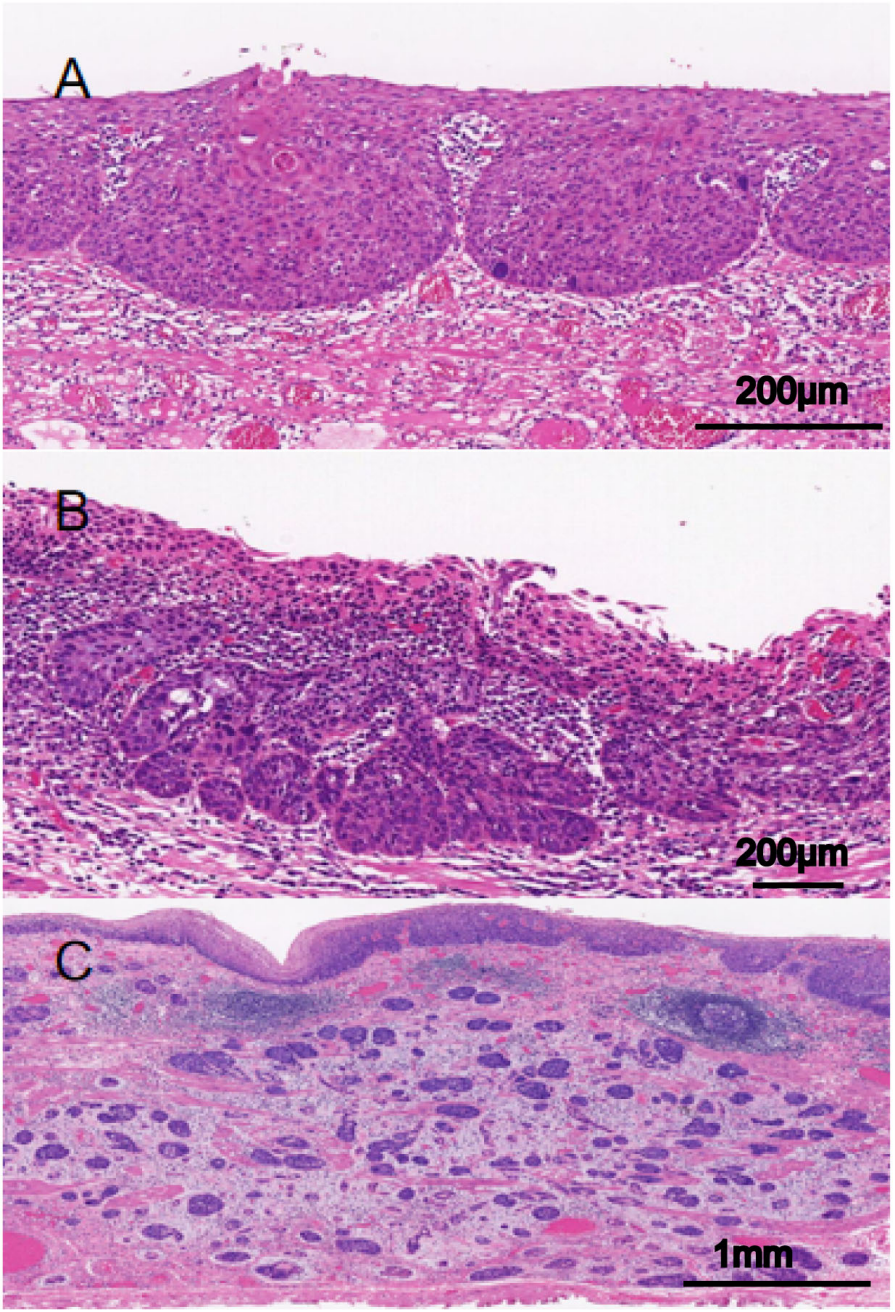
Infiltrative growth pattern (INF) of superficial esophageal squamous cell carcinoma (SESCC). **(A)** INFa (expansive): tumor nests with expansive growth and well-defined borders with surrounding tissues (The scale bar represents 200μm). **(B)** INFb (intermediate): intermediate growth pattern between INFa and INFc (The scale bar represents 200μm). **(C)** INFc, tumor nests with infiltrative growth and unclear borders with surrounding tissues (The scale bar represents 1mm).

According to the Japanese classification of esophageal cancer, the INF indicates the growth and infiltrative pattern of tumors and can be classified into one of the following three types with regard to the predominant pattern observed at the tumor margin: INFa (expansive type), expansive growth of tumor nests with a well-demarcated border from surrounding tissue; INFb (intermediate type), intermediate growth pattern between INFa and INFc; and INFc (infiltrative type), infiltrative growth of tumor nests with an ill-defined border from surrounding tissue (**Figure 1**). All these indexes were evaluated within the tumor invasive margin, which was defined as a region centered on the border separating the host tissue from tumor nests, with an extent of 1 mm.

### Evaluation of the IPCL

**Figure 2:**
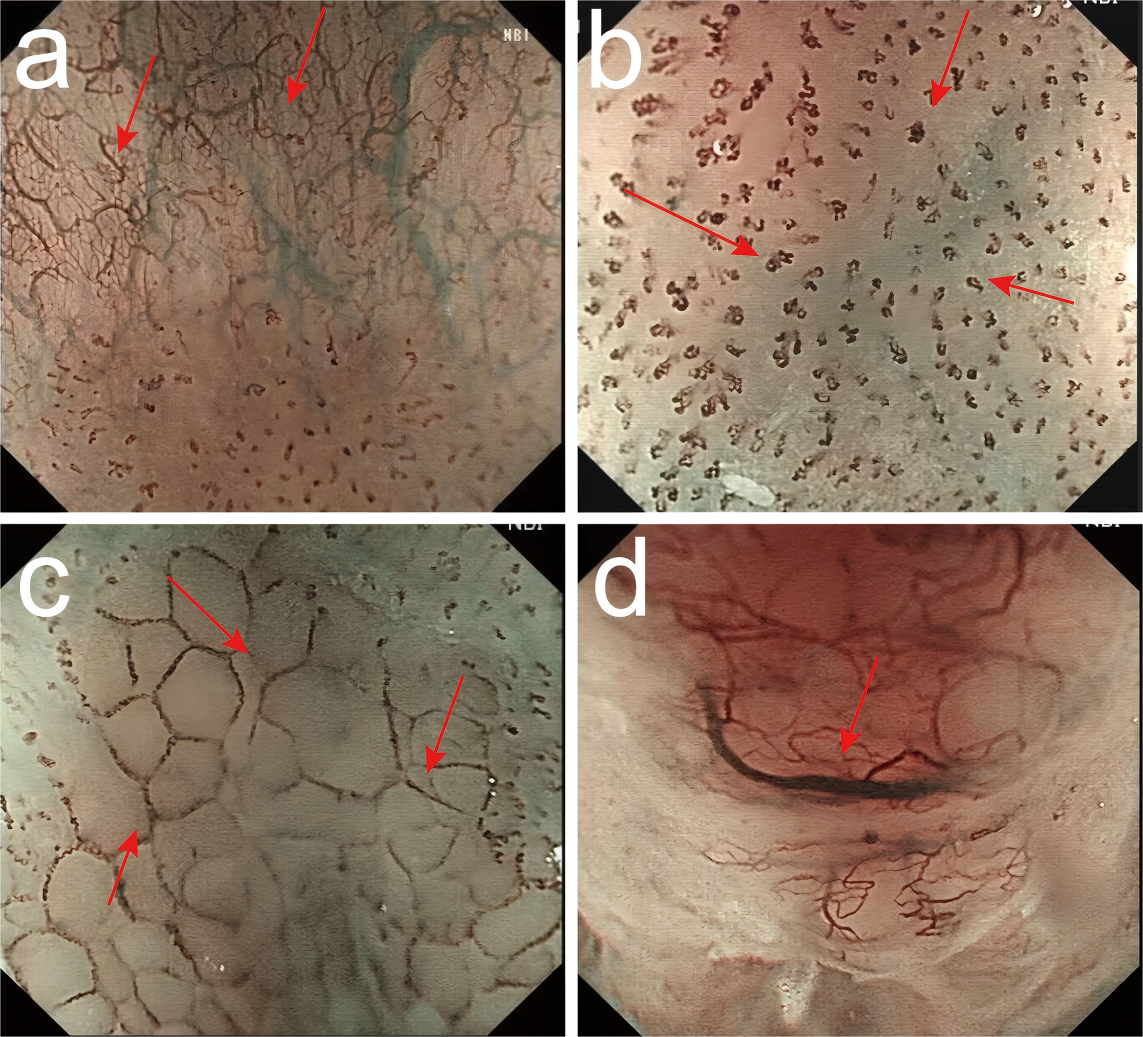
Classification of intraepithelial papillary capillary loop (IPCL) by ME-NBI. **(a)** Type A (normal): Uniform, hairpin-shaped capillaries with regular spacing and thin caliber. Mildly dilated but orderly IPCLs without distortion. **(b)** Type B1: Focal dilation of IPCLs with irregular calibers. **(c)** Type B2: Elongated, tortuous IPCLs with “tadpole-like” or “worm-shaped” morphology. **(d)** Type B3: Severely distorted, coiled, or corkscrew-shaped IPCLs with abrupt caliber changes. Partial architectural destruction (no avascular areas).

Intraepithelial papillary capillary loop (IPCL) was assessed using magnifying endoscopy with narrow-band imaging (ME-NBI) and classified as follows: Type A (normal): Uniform, hairpin-shaped capillaries with regular spacing and thin caliber. Mildly dilated but orderly IPCLs without distortion. Type B1: Focal dilation of IPCLs with irregular calibers. Type B2: Elongated, tortuous IPCLs with “tadpole-like” or “worm-shaped” morphology. Type B3: Severely distorted, coiled, or corkscrew-shaped IPCLs with abrupt caliber changes. Partial architectural destruction (no avascular areas) (**Figure 2**).

### Statistical analysis

Data were processed and statistically analyzed using SPSS 27.0. Measurement data with normal distribution, and homogeneity of variance are expressed as mean ± standard deviation (X ± S) and were compared between the groups using one-way ANOVA analysis test. Count data are described by frequency and were compared using the Pearson chi-square test, the corrected Pearson chi-square test or the Fisher’s exact test. Comparisons between groups were made using the χ² test. Multivariate logistic regression analyses were performed to identify independent distinguishing factors among INFa, INFb and INFc with odds ratios (OR) and 95% confidence intervals (95% CI) being estimated. Differences with *P*<0.05 were considered statistically significant.

## Results

### Clinicopathological characteristics analysis

The association between the INF type and clinicopathologic features of patients with SESCC is shown in **Table 1**. The 368 patients included 291 males (79.1%) and 77 females (20.9%). The mean length of the pathological lesion was 2.79 ± 1.59 cm (0.25 - 8.5 cm). Among all the cases, 42 lesions (11.4%) were located in the upper esophagus, 164 lesions (44.6%) in the middle esophagus, and 162 lesions (44.0%) in the lower esophagus. Then the postoperative retrospective analysis of endoscopic morphologic features was performed according to the 2005 Paris classification. The SESCC lesions were predominantly type of 0-II. According to the dominant morphology, there were 98 patients (26.6%) of type 0-I/IIa, 246 had 0-IIb type (66.8%), and 24 had 0-IIc/III type (6.6%).

**Table 1:** The relationship between INF and clinicopathologic features of patients with SESCC.

Variables	INFa (n=296)	INFb (n=63)	INFc (n=9)	*P*
Age (years)	63.48 ± 8.20	65.29 ± 8.929	63.56 ± 11.82	0.303
Gender, n (%)				0.651
Male	235 (79.4)	50 (79.4)	6 (66.7)	
Female	61 (20.6)	13 (20.6)	3 (33.3)	
Tumor size (cm)	2.58 ± 1.50	3.59 ± 1.58	4.04 ± 2.34	<0.001
Tumor location, n (%)				0.787
Upper	34 (11.5)	8 (12.7)	0 (0)	
Middle	130 (43.9)	28 (44.4)	6 (66.7)	
Lower	132 (44.6)	27 (42.9)	3 (33.3)	
Gross morphology, n (%)				<0.001
0-I/IIa	73 (24.7)	23 (36.5)	2 (22.2)	
0-IIb	207 (69.9)	37 (58.7)	2 (22.2)	
0-IIc/III	16 (5.4)	3 (4.8)	5 (55.6)	
Color, n (%)				0.559
Red	262 (88.5)	56 (88.9)	9 (100.0)	
Faded	34 (11.5)	7 (11.1)	0 (0)	
IPCL				<0.001
B1	246 (83.1)	27 (42.9)	1 (11.1)	
B2-Narrow	41 (13.9)	16 (25.4)	2 (22.2)	
B2-Broad	9 (3.0)	20 (31.7)	6 (66.7)	
Infiltration depth, n (%)				<0.001
M2	266 (89.9)	30 (47.6)	1 (11.1)	
M3+SM1	24 (8.1)	21 (33.3)	2 (22.2)	
SM2+SM3	6 (2.0)	12 (19.0)	6 (66.7)	
Lymphovascular invasion, n (%)				<0.001
Absence	296 (100.0)	54 (85.7)	4 (44.4)	
Presence	0 (0)	9 (14.3)	5 (55.6)	
Vertical margin, n (%)				<0.001
Negative	295 (99.7)	59 (93.7)	3 (33.3)	
Positive	1(0.3)	4 (7.3)	6 (66.7)	
Horizontal margin, n (%)				1.00
Negative	291 (98.3)	62 (98.4)	9 (100.0)	
Positive	5 (1.7)	1 (1.6)	0 (0)	

The color tones of the lesions under endoscopy were as follows: 327 patients (88.9%) showed a red tone and 41 patients (11.1%) showed a faded tone. Moreover, there were 274 patients (74.4%) with IPCL of type B1 and 94 (25.6%) with IPCL of type B2, of whom 59 patients (62.8%) had the B2-Narrow type and 35 (37.2%) had the B2-Broad type. There were 297 patients (80.7%) with M2, 47 patients (12.8%) with M3+SM1, and 24 patients (6.5%) with SM2+SM3. Furthermore, there were 14 patients (3.8%) with lymphovascular infiltration. Six patients (1.6%) had a positive horizontal resection margin, and 14 patients (3.8%) had positive vertical resection margin.

### Statistical analysis of INFa, INFb, INFc and clinicopathological parameters of SESCC patients

As shown in **Table 1**, all the lesions were stratified into three groups based on INF types, there are no significant statistically differences in age, gender, tumor location, color, and horizontal margin among the three groups (All *P* > 0.05). Nevertheless, the INF of tumor was associated with tumor size, gross morphology, IPCL, depth of tumor invasion, lymphovascular invasion, vascular invasion and vertical positive margins (All *P* < 0.05). INFc-type SESCC was significantly associated with larger lesion size, IPCL, and depressed morphology (0-IIc/III). In addition, compared with INFa and INFb, INFc type SESCC exhibited deeper infiltration depth and is more likely to have lymphovascular and vascular invasion and the increased vertical margin positivity.

### Independent distinguishing factors among INFa, INFb and INFc

In order to further analyze the independent distinguishing factors of INF types, the significant univariate analysis of clinical characteristics in **Table 1** were included in the multivariate logistic regression analysis (**Table 2**). INFa served as the reference group, and the results showed that tumor size (β=0.28, *P*=0.004; OR=1.32, 95%CI:1.09-1.59), IPCL type B (β=0.81, *P*=0.004; OR=2.24, 95%CI:1.30-3.85), and infiltration depth (β=0.81, *P*=0.017; OR=2.24, 95%CI:1.15-4.35) were significantly associated with INFb. In contrast, the correlation between INFc and lymphatic vessel invasion was significantly enhanced (β=8.77, *P*=0.007; OR=6456.93, 95% CI: 10.96-3803785.49). And simultaneously, lymphatic vessel invasion was an independent risk factor for INFc.

**Table 2:** Multivariate logistic regression analysis of the clinicopathological characteristics among INFa, INFb and INFc.

Variables	INFb					INFc				
	*β*	SE	*P*	OR	95%CI	*β*	SE	*P*	OR	95%CI
Tumor size (cm)	0.28	0.1	0.004	1.32	(1.09-1.59)	-0.49	0.57	0.387	0.61	(0.20-1.86)
Gross morphology						-0.03	1.23	0.98	0.97	(0.09-10.72)
IPCL	0.81	0.28	0.004	2.24	(1.30-3.85)	1.02	0.93	0.27	2.78	(0.45-17.17)
Infiltration depth	0.81	0.34	0.017	2.24	(1.15-4.35)	2	1.41	0.157	7.39	(0.46-117.80)
Lymphovascular invasion	2.07	1.14	0.069	7.93	(0.85-73.94)	8.77	3.25	0.007	6456.93	(10.96-3803785.49)
Vertical margin	-0.1	1.02	0.923	0.91	(0.12-6.64)	3.57	2.69	0.185	35.69	(0.18-7008.40)

## Discussion

Esophageal cancer (EC) is prone to early lymph node metastasis and is associated with poor prognosis and high mortality. Early detection of esophageal cancer and appropriate selection of treatment modalities are crucial for reducing the mortality rate and improving the outcomes of patients with esophageal cancer (1, 2). Infiltrative growth pattern (INF) has been reported to be a prognostic factor for stomach, gallbladder, and bladder cancer (7–10). In this study, we focused on the tumor INF type and clinicopathological characteristics of superficial esophageal squamous cell carcinoma (SESCC). Our study found that the tumor INF types were significant correlation with the size, gross morphology, intraepithelial papillary capillary loop (IPCL), depth of tumor invasion, lymphovascular invasion, and positive vertical resection margins. Furthermore, endoscopic examination shows that SESCC is more likely to exhibit INFc with increasing tumor size, gross morphology of superficially depressed (IIc) and the presence and morphology of IPCL type B2.

Early detection is crucial for improving the outcomes of patients with EC. With advancements in endoscopic equipment and techniques, such as the advent of ME-NBI, it is now easier to detect esophageal cancer at an early stage and improve patient outcomes. Yorimitsu et al. concluded when type B2 blood vessels were present, INFb and INFc lesions were associated with substantially increased risk of lymphatic infiltration (11). Therefore, careful visualization of the lesion using NBI-ME prior to ESD is essential for identifying type B2 vessels and for predicting histopathological features. Tanaka et al. (12) retrospectively analyzed 78 patients with B2 lesions, the ROC curve analysis showed that the optimal cut off for the B2 target area was 4 mm, The sensitivity, specificity, positive predictive value, and negative predictive value of B2-broad in predicting M3 or deeper infiltration were enhanced. The diagnostic accuracy of type B2 was improved by evaluating the area of type B2. Therefore, we classified the 368 patients with SESCC according to the JES IPCL classification criteria and analyzed the correlation between INF and IPCL patterns. The IPCL was predominantly type B1 vessel for INFa lesions, and predominantly type B2 vessel for INFb lesions, of which 16 patients were B2-narrow and 20 patients were B2-broad. The frequency of type B2-broad vessels (6/9, 66.7%) for INFc lesions was likely attributed to the disruption of the IPCL (11). Our study of multivariate analysis results showed that, IPCL was identified as an independent risk factor for INFb. Therefore, endoscopic examination of the IPCL pattern is useful for predicting histopathological features, such as depth of infiltration and INF type, and guiding preoperative treatment plans.

The tumor cell infiltration depth has been shown to be significantly associated with lymph node metastasis and tumor prognosis (13–15). In agreement, our results also indicate that there is a significant correlation between infiltration depth and INF of SESCC. The depth of infiltration was mostly M2 in patients with INFa, M2 and M3+SM1 in patients with INFb, and SM2+SM3 in patients with INFc. Furthermore, significant relationships have been identified between infiltration depth and lymph node and hematogenous metastases, indicating that carcinoma *in situ* has lower rates of lymph node and hematogenous metastases and better prognosis, while submucosal carcinoma has a higher rate of metastasis and poorer prognosis. Furthermore, our data revealed the correlation between INF types and infiltration depth in SESCC. INFc-type lesions, which exhibit the highest invasiveness, showed significantly deeper infiltration compared to INFa/b types. In our multivariate analysis, infiltration depth was identified as an independent risk factor for INFb. These findings suggest that INFc-type SESCC may have a higher metastatic potential and worse clinical outcomes.

Additionally, INFc tumors have been reported to have a high risk of lymph node metastasis as the infiltration depth increases (16). In addition, studies on the correlation between INF and prognosis of gastric, colon, and gallbladder cancers have shown no significant differences in prognosis between patients with INFa and INFb lesions, but significantly lower survival rates and worse outcomes have been identified among patients with INFc lesions (17–19). This difference was attributed to the higher rates of lymph node metastasis and vessel infiltration in patients with INFc lesions (10). According to a Japanese study, submucosal gastric cancer cells with INFc can lead to patient outcomes similar to those in the progressive stage (20). Morikawa et al. reported that in stage I-III colon cancer, tumor cells with INFc predict a worse prognosis (10). Other researchers have combined INF typing and TNM staging (including T stage and lymph node metastasis) to determine the prognosis of patients with upper tract urothelial cancer and guide clinical treatment (21). In our study, lymphovascular invasion rates were 14.29% (INFb) versus 55.56% (INFc), underscoring a robust correlation between INF subtype and vascular infiltration in SESCC. In the multivariate analysis of **Table 2**, lymphovascular invasion was identified as an independent risk factor for INFc. These findings collectively suggest that INFc-type tumors, characterized by heightened cellular invasiveness, promote deeper tissue infiltration and lymphovascular invasion, thereby increasing metastatic potential and worsening prognosis.

Jin et al. (22) retrospectively analyzed the relationship between the INF and tumor immune microenvironment of 593 patients with stage T1 ESCC, along with their predictive value for lymph node metastasis and overall survival, and found that INFc was an independent risk factor for lymph node metastasis. Furthermore, INFc and low-grade tumor infiltrating lymphocytes (TILs) were independent predictors for poor outcomes; INFc was associated with immunosuppression; and INF combined with TILs was useful for improving the prognosis of patients with ESCC.

In previous studies, INF is assessed at the deepest tumor infiltration margins, which are only present in surgical specimens and cannot be assessed with biopsy specimens. Therefore, INF assessment cannot be used for the preoperative selection of treatment strategy. Kanda et al. reported that INFc was associated with a high rate of peritoneal recurrence in gastric cancer, while the INFa or INFb type was associated with a high rate of liver recurrence (9). INFc has also been associated with recurrence in bladder and colorectal cancers. Therefore, they believe that INF subtype may facilitate the selection of the most appropriate postoperative treatment (23–28). However, in this study, we focused on the tumor INF type and clinicopathological characteristics in SESCC. We found that the tumor diameter, macroscopic type, and IPCL under endoscopy, the INF of the tumor can be roughly predicted, which helps to improve the accuracy of biopsy and achieve the goal of guiding the rational selection of surgical methods. Unlike existing methods that often provide diagnostic information only after resection, INF typing allows for earlier identification of high-risk lesions (particularly INFc) during initial endoscopy, enabling more timely treatment decisions. In additionally, the INF type can be used for histopathologic evaluation of endoscopically resected specimens, to assess the likelihood of metastasis and the need for any additional treatment.

This study has some limitations. Firstly, this was a single-center study with only 368 patients, resulting in a relatively small number of patients with INFc lesions after grouping, which possibly impacting the study results and leading to the absence of statistical significance between group comparisons. Secondly, there may also be potential biases in the article, such as the heterogeneity of endoscopic assessment which might affect the results. Despite these limitations, our research findings are consistent with previous studies that have linked INFc with aggressive phenotypes in other cancers. Hence, further large-cohort multicenter studies are warranted to confirm the generalizability of our findings. In conclusion, INF is associated with tumor size, macroscopic type, IPCL, depth of tumor invasion, lymphovascular invasion, and positive vertical resection margins. In addition, multivariate logistic regression analysis shows that tumor size, IPCL, and infiltration depth were significantly associated with INFb. Lymphatic vessel invasion was an independent risk factor for INFc. Our findings may help improve the endoscopic prediction of INF and tumor status and guide the preoperative surgical approach and subsequent postoperative treatments.

## Conclusion

In the study of SESCC, we found a significant correlation between tumor infiltrative growth pattern (INF) and diameter, macroscopic type, intraepithelial papillary capillary loop (IPCL), depth of tumor invasion, lymphovascular invasion, vertical positive margins. And simultaneously, tumor size, IPCL and infiltration depth are significantly correlated with INFb, as well as lymphatic invasion is an independent risk factor for INFc. Above all, careful endoscopic visualization of tumor size, IPCL, and gross morphology can improve the prediction of INF and tumor status, facilitating informed preoperative selection of surgical approach and subsequent postoperative treatments.

## Data Availability

The original contributions presented in the study are included in the article/supplementary material. Further inquiries can be directed to the corresponding authors.
